# Fabrication of AIE Polymer-Functionalized Reduced Graphene Oxide for Information Storage

**DOI:** 10.3390/molecules28176271

**Published:** 2023-08-27

**Authors:** Kai Gao, Wei Li, Xiaoyang Wang, Sai Sun, Bin Zhang

**Affiliations:** 1 Sinopec Shanghai Research Institute of Petrochemical Technology, Shanghai 201208, China; 2 Key Laboratory for Advanced Materials and Joint International Research Laboratory of Precision Chemistry and Molecular Engineering, School of Chemistry and Molecular Engineering, East China University of Science and Technology, Shanghai 200237, China; 3 Guangxi Key Laboratory of Information Material, Engineering Research Center of Electronic Information Materials and Devices, School of Material Science and Engineering, Guilin University of Electronic Technology, Guilin 541004, China

**Keywords:** aggregation-induced emission, reduced graphene oxide, surface modification, information storage

## Abstract

Reduced graphene oxide (RGO) has been extensively studied and applied in optoelectronic systems, but its unstable dispersion in organic solvents has limited its application. To overcome this problem, the newly designed and developed aggregation-induced emission (AIE) material poly[(9,9-bis(6-azidohexyl)-9H-fluorene)-alt-(9-(4-(1,2,2-triphenylvinyl)phenyl)-9H-carbazole)] (PAFTC) was covalently grafted onto RGO to produce (PFTC-*g*-RGO). The solubility of two-dimensional graphene was improved by incorporating it into the backbone of PAFTC to form new functional materials. In resistive random access memory (RRAM) devices, PFTC-*g*-RGO was used as the active layer material after it was characterized. The fabricated Al/PFTC-*g*-RGO/ITO device exhibited nonvolatile bistable resistive switching performances with a long retention time of over 10^4^ s, excellent endurance of over 200 switching cycles, and an impressively low turn-ON voltage. This study provides important insights into the future development of AIE polymer-functionalized nanomaterials for information storage.

## 1. Introduction

Graphene was first mechanically exfoliated by K. S. Novoselov and A. K. Geim [1]. It has attracted considerable attention from both experimental and theoretical studies due to its unique structure and outstanding physical properties. Graphene is a one-atom-thick, two-dimensional (2D) pseudo-infinite carbon material comprising sp2-hybridized aromatic carbon. It is characterized by a bulky surface area, excellent mechanical strength, and high charge/hole mobility. In addition, it has outstanding thermal and electrical conductivities [2,3,4]. Graphene has been considered a promising material for supercapacitors, solar cells, field-effect transistors, and optical limiters [5,6,7,8]. In recent years, graphene-based materials have received remarkable attention from researchers. Most studies on graphene mainly focus on the chemistry of graphene oxide (GO) with reactive oxygen functionalities, including carboxylic acid groups at the sheet edges of GO and epoxy and hydroxyl groups on the basal planes [9,10]. However, these functional groups are thermally unstable and can impede the electron/hole mobilities, thus retarding the charge carrier mobility. In addition, the hybridization state of sp2/sp3 in the aromatic layer of GO hampers the electrical conductivity and electron/hole mobilities compared to pristine graphene [11,12]. RGO can be obtained by reducing GO. Compared to GO, the removal of oxygen-containing groups can partially recover the ballistic transport and charge carrier mobility and restore the sp2 π-conjugated hybridization of pristine graphene to a large degree [13,14,15].

Despite the excellent properties and prospects of graphene, RGO sheets aggregate as multilayer structures or agglomerates due to van der Waals forces and intense π–π stacking interactions, which renders it with poor solubility or dispersion stability in both aqueous and organic solvents, thereby limiting its practical applications [16,17,18,19]. To improve the dispersion ability and stability of RGO, researchers have devoted considerable efforts to exploring the chemical modification of RGO, such as noncovalent functionalization and covalent functionalization [20,21]. Over the past decade, phthalocyanine, porphyrins, carbazole, azobenzene, polymers, nanoparticles, and ionic complexes were successfully introduced into the preparation of functional graphene derivatives [22,23,24,25].

To reduce the π–π stacking interactions, steric hindrance can be introduced into the preparation of functional graphene derivatives to suppress the interlayer cohesive energies, separating RGO sheets from each other. Generally, luminogens with aggregation-induced emissions (AIEgens) undergo stereospecific hindrance due to their physical constraints, making them nonluminescent or weakly luminescent in solutions but highly emissive in the aggregation state [26]. Tetraphenylethylene (TPE) derivatives with the restriction of intramolecular rotations (RIR) as a working mechanism are considered iconic AIEgens. They have a wide range of practical applications in optoelectronic systems, including OLEDs, OFETs, self-assembly systems, and photovoltaics [27,28,29,30]. Recently, we synthesized a novel material poly[(9,9-dihexyl-9H-fluorene)-alt-(9-(4-(1,2,2-triphenylvinyl)phenyl)-9H-carbazole)]-RGO (PFTC-g-RGO) in which the poly[(9,9-bis(6-azidohexyl)-9H-fluorene)-alt-(9-(4-(1,2,2-triphenylvinyl)phenyl)-9H-carbazole)] (PAFTC) polymer was used to covalently functionalize RGO. 

Although numerous studies on graphene-like low-dimensional nanomaterial-based memory devices have been reported [31,32,33,34,35,36], the use of AIE polymer-functionalized RGO materials for nonvolatile RRAMs has not been reported. In this work, the covalent grafting of AIE polymer was achieved via the RGO surface using reduced oxidized graphene as a two-dimensional template through the reaction of azide groups with double bonds. The generated material, PFTC-g-RGO, was used to produce a memory device. The manufactured device with the Al/PFTC-g-RGO/ITO structure exhibited high ON/OFF ratios (>10^3^), long retention times, outstanding durability, a repetitive “write-read-erase-read-write” capability, and a nonvolatile bistable resistive switching behavior at voltages of +0.60 V (writing) and −2.30 V (erasing). As shown in Table 1, our synthesized material had a lower turn-ON value than similar materials reported in previous works, indicating that the device required less voltage to perform write operations. AIE-active polymer-functionalized RGO provides a new avenue for designing memory devices.

## 2. Results and Discussion

**Scheme 1:**
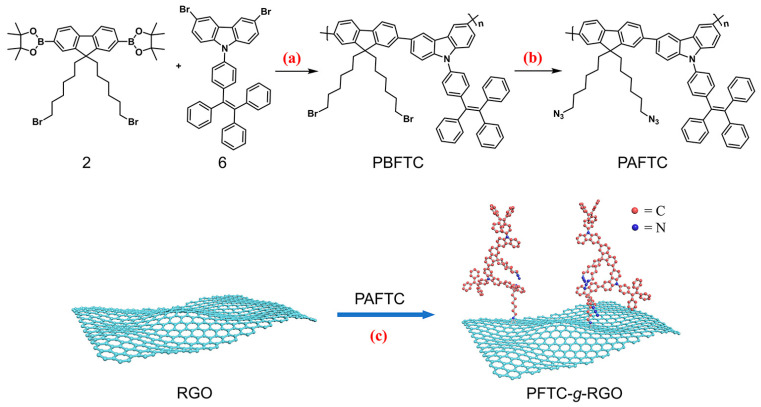
Synthesis of PAFTC and PFTC-*g*-RGO using (**a**) Pd(PPh_3_)_4_, K_2_CO_3_, toluene, and water at 85 °C for 48 h; (**b**) NaN_3_, DMF, THF, and RT for 48 h; (**c**) NMP at 160 °C for 3 d.

In this study, PAFTC, an AIE polymer that was developed and synthesized, was directly connected with RGO in the presence of N-methylpyrrolidone (NMP) to produce the soluble derivative of poly[(9,9-dihexyl-9H-fluorene)-alt-(9-(4-(1,2,2-triphenylvinyl)phenyl)-9H-carbazole)] (PFTC). As shown in Scheme 1, PFTC increased the solubility of RGO by covalently functionalizing RGO with the conjugated polymer PAFTC, enabling the fabrication of RGO-based thin film devices using methods that may be processed in a solution.

**Figure 1:**
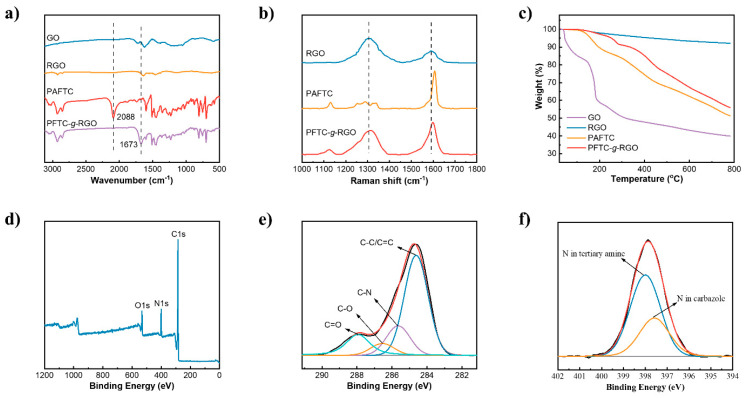
(**a**) IR spectra of GO, RGO, PAFTC, and PFTC-*g*-RGO. (**b**) Raman spectra of the samples (λ_ex_ = 785 nm). (**c**)TGA curves of GO, RGO, PAFTC, and PFTC-*g*-RGO. (**d**) XPS wide scan spectra of PFTC-*g*-RGO, (**e**) C1s, and (**f**) N1s core-level spectra of PFTC-*g*-RGO.

The effective covalent functionalization of RGO with PAFTC via nitrene chemistry was corroborated with IR spectroscopy (Figure 1a). GO exhibited several characteristic peaks at 1056, 1720, and 1384 cm^−1^, corresponding to the stretching vibration of C-O from the hydroxyl groups, the stretching vibration of C=O, and the deformation vibration of O-H from the carbonyl and carboxyl groups, respectively. The characteristics peaks of 1623 and 1143 cm^−1^ corresponded to the C=C stretching of the graphitic domains and the C-O stretching of the epoxy groups, respectively. In the spectra of RGO, the characteristic peaks of GO almost disappeared, indicating the successful reduction of GO. PAFTC exhibited three characteristic peaks. The peaks at 2927 and 2854 cm^−1^ were assigned to the asymmetric and symmetric stretching vibrations of the methylene groups. The peak at 1461 cm^−1^ was assigned to the deformation vibration of the methylene groups. A strong absorption peak located at 2088 cm^−1^ corresponded with the stretching vibration of the azido groups. After covalent modification, the peak of the azido groups disappeared, and a new absorption band centered at 1673 cm^−1^ emerged, which was related to C=C-H stretching in the RGO, indicating the covalent functionalization of the RGO with PAFTC via nitrene chemistry was successful. The Raman spectra of RGO (Figure 1b) with a 785 nm laser excitation revealed two distinct bands at 1306 (D band) and 1591 (G band) cm^−1^ at an intensity ratio (I_D_/I_G_) of 2.05. The G band was utilized to show the extent of RGO alteration, and the D band was used to detect the covalent modification process that changed sp^2^ sites to sp^3^ [37]. The D and G bands were red-shifted to 1315 and 1598 cm^−1^, respectively, after the PFTC was covalently grafted onto the RGO surface. The D band and G band intensity ratios were reduced to 0.75. The Raman spectra revealed that the functionalized RGO had a lower I_D_/I_G_ ratio than pure RGO. This result showed that the prepared polymer was successfully covalently grafted onto RGO.

Thermogravimetric analysis (TGA) was conducted under nitrogen protection at a temperature ranging from 35 to 800 °C to determine the successful reduction of GO and estimate the RGO content in the final product. The spectrum (Figure 1c) of GO revealed that the GO was thermally unstable. After the GO was heated to 100 °C, it suffered a 13.4% weight loss, which was ascribed to the evaporation of the absorbed water. The main weight loss of 35.1% occurred at a temperature ranging from 100 to 300 °C, and the 35.1% weight loss can be attributed to the decomposition of oxygen-containing groups. At 800 °C, the weight loss of 12.0% was caused by the more stable oxygen functionalities. RGO exhibited a slower and more stable TGA curve than GO, confirming the successful reduction of GO. Due to the removal of residual oxygen functionalities, RGO had a weight loss of 4.7% at a temperature range of 100–300 °C. In addition, a 6.6% weight loss was observed as the temperature increased to 600 °C. PAFTC exhibited two weight loss platforms ranging from 100 to 300 °C (8.8%) and 300 to 600 °C (23.8%). After the modification of the PAFTC, the curve of PFTC-*g*-RGO was less than that of PAFTC, with 9.2% and 26.7% weight losses ranging from 100 to 300 °C and 300 to 600 °C, respectively. At temperatures ranging from 100 to 800 °C, the total weight losses of the RGO, PAFTC, and PFTC-*g*-RGO were 14.0, 45.0, and 42.2%. By assuming that the PAFTC residue remaining in the PFTC-*g*-RGO at 800 °C had the same weight percentage as that of the PAFTC, we estimated the content of RGO in the PFTC-*g*-RGO as 16.8% (14.0 + 45.0–42.2%).

Three indications of oxygen/carbon/bromine functionalities were visible in the wide scan XPS spectrum of the PFTC-*g*-RGO (Figure 1d) at energies of 531.1 (O1s), 400.1 (N1s), and 285.1 (C1s). The peaks of the carbon functionalities were observed at 284.6 (C-C/C=C), 285.6 (C-N), 286.5 (C-O), and 287.9 (C=O) eV in accordance with the PFTC-*g*-RGO C1s core-level XPS spectrum (Figure 1e). In the N1s core-level XPS spectra of the PFTC-*g*-RGO (Figure 1f), the signals at 399.6 and 400.0 eV were ascribed to the nitrogen in the C-N bonds of the carbazole and the tertiary amine, respectively. These results showed that the PFTC was successfully grafted onto the RGO surface.

**Figure 2:**
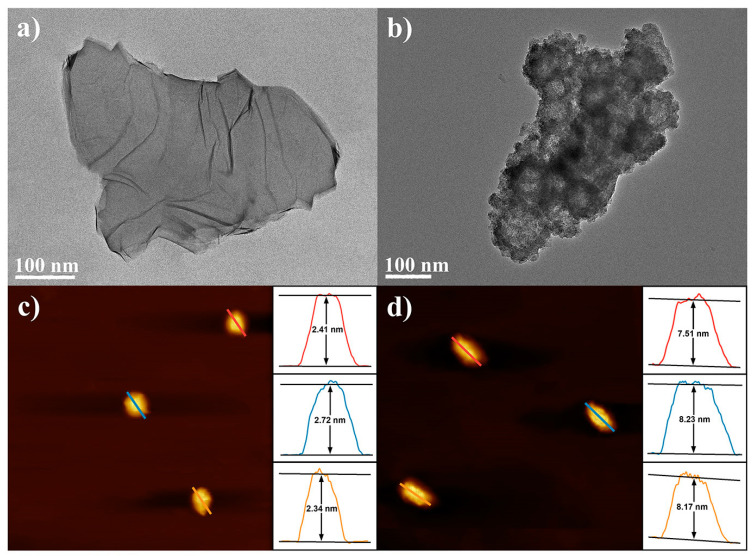
TEM images of (**a**) RGO and (**b**) PFTC-*g*-RGO. AFM images (scanning size: 4 × 4 μm^2^) of (**c**) RGO and (**d**) PFTC-*g*-RGO.

The morphology of RGO and PFTC-*g*-RGO was further understood through TEM and AFM measurements. By comparing the morphologies in Figure 2a,b, the transparency of the covalently modified nanosheets decreased, and the previously visible folds and wrinkles were no longer visible. As depicted in Figure 2c, the highly transparent RGO sheets exhibited an average thickness of 2.49 nm with a lateral dimension size of approximately 400 nm. Following the covalent modification of the RGO via nitrene chemistry (Figure 2d), the average thickness of the functionalized RGO increased to 7.97 nm, suggesting the presence of a polymer layer on the surface of the RGO. It is speculated that the average size of the polymer was 5.48 nm.

**Figure 3:**
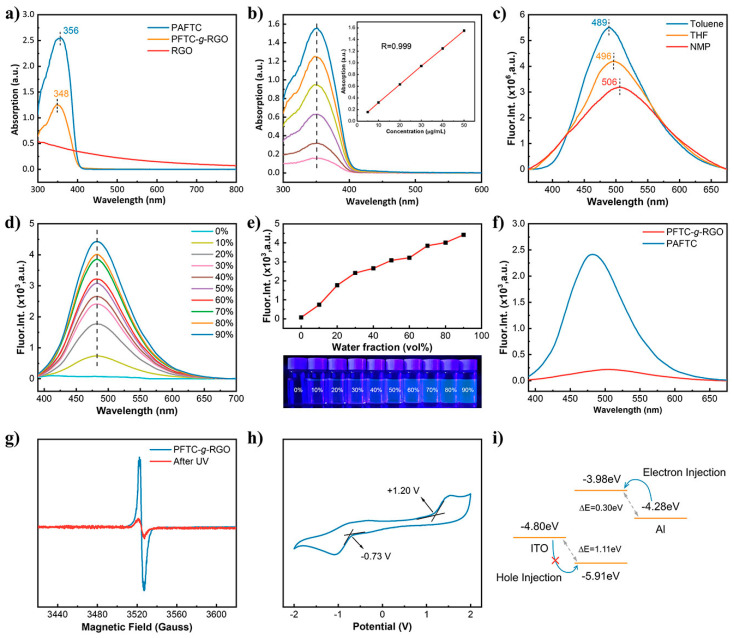
(**a**) UV/Vis spectra of RGO, PAFTC, and PFTC-*g*-RGO. (**b**) UV/Vis spectra of PFTC-*g*-RGO at a range of concentrations from 5 to 50 μg∙mL^−1^. Inset shows the correlation curve of PFTC-*g*-RGO at 350 nm to the concentration. (**c**) Photoluminescence spectra of PFTC-*g*-RGO in different solvents (λ_ex_ = 350 nm). (**d**) Photoluminescence spectra of PAFTC in a solvent mixture of NMP and H_2_O with different water contents (0→90%). (**e**) Statistical graph and a digital photograph of the fluorescence intensity of the sample in different water contents. (**f**) Photoluminescence spectra of PAFTC and PFTC-*g*-RGO in a solvent mixture of NMP and H_2_O with 30% water content. (**g**) Electron paramagnetic resonance (EPR) spectra of PFTC-*g*-RGO before and after 450 nm laser illumination. (**h**) Cyclic voltammograms of the PFTC-g-RGO film with an aqueous solution of tetrabutylammonium hexafluorophosphate (0.05 M) as the supporting electrolyte, Ag/AgCl as the reference electrode, and a scan rate of 100 mV/s. (**i**) Abridged general view of charge injection processes in the memory device.

The UV/Vis absorption spectra of GO, PAFTC, and PFTC-*g*-RGO are illustrated in Figure 3a. The PAFTC polymer was characterized by a maximum absorption peak at 356 nm and a tiny shoulder peak at 318 nm, which was assigned to π–π* transitions of highly delocalized π electrons. In the spectra of PFTC-*g*-RGO, the peak of PAFTC at 356 nm was blue-shifted to 348 nm, indicating an electronic interaction between the RGO and PAFTC. To investigate the solubility of PFTC-*g*-RGO, we also measured the absorption (Figure 3b) at different concentrations in NMP. The absorption intensities at 350 nm were plotted against the concentrations. The results showed that the absorption intensities were linearly correlated with the concentrations of PFTC-*g*-RGO. According to the Lambert–Beer law, the correlation coefficient was 0.999, indicating the excellent dispersion stability of PFTC-*g*-RGO in organic solvents.

When a molecule with a donor–acceptor structure is photoexcited, a photoinduced electron transfer is the straightforward process in which an electron is transferred from an electron-donating species to an electron-accepting species, resulting in the radical cation of the donor and the radical anion of the acceptor [38]. We utilized steady-state fluorescence measurements and EPR technology to study the photoinduced intramolecular events occurring in PFTC-g-RGO consisting of PFTC donor and RGO acceptor units. The fluorescence spectra of PFTC-g-RGO in toluene exhibited a prominent emission peak at 489 nm (Figure 3c). The position of this intense emission peak shifted from 489 nm in toluene to 496 nm in tetrahydrofuran and finally to 506 nm in N-methyl-pyrrolidone as the polarity of the organic solvents increased. Concurrently, there was a gradual decrease in fluorescence intensity. These findings suggest that the fluorescence quenching process may be attributed to the electron transfer mechanism from PFTC to RGO*.

PAFTC exhibited standard AIE characteristics as predicted (Figure 3d). PAFTC did not exhibit an NMP emission peak under the illumination of a 350 nm laser. As shown in Figure 3e, the PAFTC polymer emitted cyan fluorescence at 482 nm when water was added to the solvent, and the intensity of the emission steadily increased with increasing water content. The fluorescence intensity was continuously increased as the water content approached 90%. AIE activity was observed in PFTC-*g*-RGO in contrast to PAFTC. When the PAFTC was covalently grafted onto the surface of the RGO, the fluorescence intensity of the PAFTC was significantly reduced (Figure 3f). About 91.32% of the fluorescence intensity of the PAFTC film was reduced due to the electron transfer from the PFTC to the RGO, resulting in PFTC^+^-RGO^−^ radical-ion pairs. The EPR spectra of PFTC-*g*-RGO reveal a characteristic radical signal (Figure 3g) before illumination, indicating the presence of unpaired electrons or free radicals in the PFTC-*g*-RGO system. The signal strength significantly decreased after the irradiation of PFTC-*g*-RGO with a 450 nm laser for approximately 30 s under ambient lighting. This phenomenon is mainly attributed to the photoinduced intramolecular electron transfer effect of the PFTC covalently grafted RGO system.

The beginning oxidation/reduction potentials (Eox/Ered) were observed to be at +1.20/−0.73 V against Ag/Ag^+^, which corresponded to +1.49/−0.44 V vs. the saturated calomel electrode (SCE) from the cyclic voltammogram curve of the PFTC-*g*-RGO film in Figure 3h. The electron affinity (EA), the ionization potential (IP), and the HOMO/LUMO energy levels of PFTC-*g*-RGO were determined using reported equations in the literature [31]. Therefore, the HOMO, LUMO, IP, EA, and bandgap values of PFTC-*g*-RGO were predicted to be −5.91, −3.98, 5.88, 3.95, and 1.93 eV, respectively. Given the calculated energy bands, the energy level structure of the memory device was plotted (Figure 3i). The band gap between the work function of ITO (−4.80 eV) and the HOMO of PFTC-*g*-RGO was 1.11 eV. However, the band gap was only 0.30 eV between the LUMO energy level and the work function of Al (−4.28 eV). The difference observed in the latter and former band gaps indicated that the conducting mechanism in PFTC-*g*-RGO was dominated by electrons.

**Figure 4:**
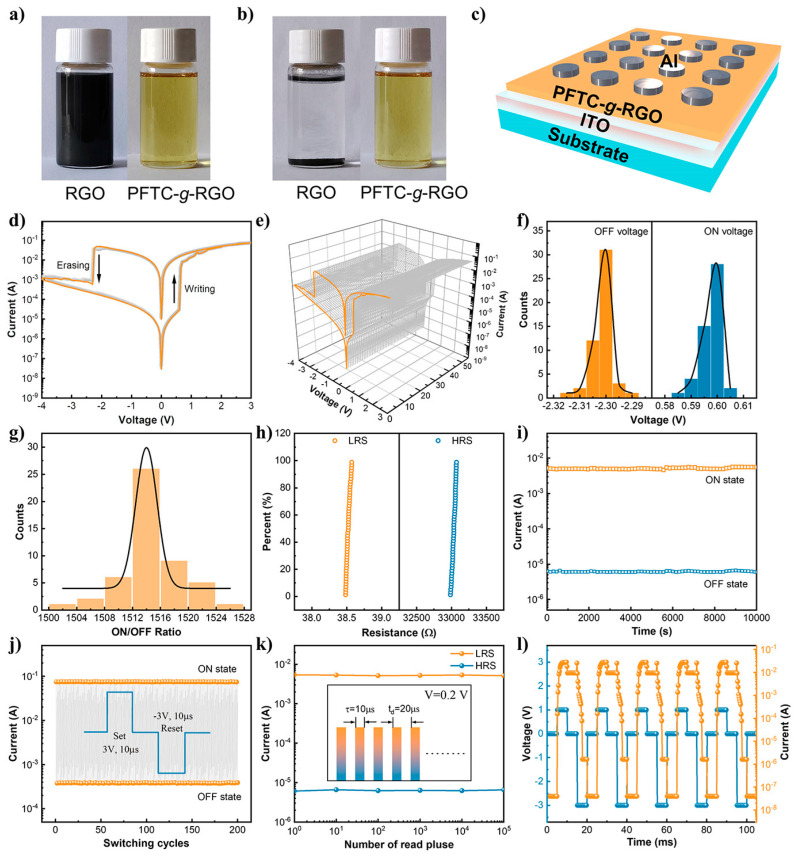
Digital photographs of the samples dispersed in NMP: (**a**) the samples dispersed immediately after sonication and (**b**) the samples rested for 24 h after sonication. The concentration of each sample was 5 mg/mL. (**c**) An illustration of a memory device schematic. (**d**) The current–voltage performance of the preparative device. (**e**) Three-dimensional I-V performance chart. (**f**) Distribution histogram of the device at OFF and ON voltages. (**g**) The distribution histogram presents the ON/OFF ratio of the device. (**h**) Weibull distribution of the resistance in the low resistance and high resistance states at +0.2 V. (**i**) Retention times of the device at +0.2 V in both the ON and OFF states. (**j**) Al/PFTC-*g*-RGO/ITO device operating with switched voltages of ±3 V at a pulse width of 10 μs and a period of 40 μs. (**k**) Time stability of the ON and OFF states of the Al/PFTC-*g*-RGO/ITO device under +0.2 V at a pulse width of 10 μs and a period of 20 μs. (**l**) The current–time response of the device during the write–read–erase–read–write cycles.

As shown in Figure 4a, the RGO was almost uniformly dispersed in the solution, while PFTC-*g*-RGO completely dissolved. After the two solutions were transferred to bottles for 24 h (Figure 4b), the RGO was suspended, and sedimentation was distributed at the upper and lower layers of the solution. No significant change was observed in the solution of PFTC-*g*-RGO. The digital photographs revealed that the solubility of RGO in NMP was greatly enhanced by grafting the AIE polymer onto the surface of the RGO. In the memory device configuration (Figure 4c), a thin layer of the synthetic polymer was spin-coated between an indium–tin oxide (ITO) bottom electrode and a vacuum-deposited spherical aluminum top electrode. The current voltage characteristics of the PFTC and PFTC-g-RGO-based memory devices demonstrated memory behavior. The conductivity state of the fabricated device quickly switched from the OFF state (10^−5^ A) to the ON state (10^−2^ A) at a threshold voltage of +0.60 V (Figure 4d), indicating a successful writing process. This behavior observed in the digital information storage device is relatively similar to expectations. As expected, the device remained in the ON mode during the subsequent forward sweep. Although the electrical power supply was turned off, the ON mode remained unchanged. This characteristic suggests that the memory performance of the device is nonvolatile, meaning that the computer’s memory system can retain important data even without an electrical power supply. The ON state returned to its original OFF state when a negative sweep voltage of 0 to −3 V was applied to the device. This was evident from the rapid decrease in current from 10^−2^ to 10^−5^ A at the threshold voltage of −2.30 V. This process is referred to as the erasure process. The conductivity of the device remained low during the subsequent backward sweep. Subsequently, the OFF mode was reprogrammed to the ON state after applying a positive sweep. These findings indicate that the designed electronic device could potentially function as a nonvolatile rewritable memory with an ON/OFF current ratio exceeding 10^3^.

To successfully add polymer to silicon or replace the silicon cell in devices, polymer memory devices must meet a few requirements: a high ratio of OFF to ON current, repetitive write–read–erase–read–rewrite capability, and device stability. At a read voltage of +0.60 V, a high ON/OFF current ratio of over 10^3^ was achieved (Figure 4d). In digital memory processes, a high ON/OFF current ratio might lead to a low misreading rate. For the Al/PFTC-*g*-RGO/ITO device, further statistical data were collected by measuring hundreds of different top aluminum electrodes in one memory cell. This measurement was conducted to evaluate the repetitive write–read–erase–read–rewrite capabilities of the device. Figure 4e displays more than 50 highly repeatable memory I-V loops. Several top aluminum electrodes displayed outstanding nonvolatile flash-type memory properties. Figure 4f shows the probability histogram distributions of the “turn ON” and “turn off” voltage at various top aluminum electrodes. An extremely narrow range between 0.585 and 0.605 V was observed in the “turn ON” voltage, while the “turn off” voltage had an extremely narrow range between −2.315 and −2.290 V, indicating strong reproducibility. At a read voltage of +0.60 V, a high ON/OFF current ratio of >10^3^ was achieved (Figure 4g). After the switching ratio data were analyzed, a Gaussian fit was performed. The fitted curve indicates that the data are evenly distributed, and the switching ratio remains stable. The Weibull distribution (Figure 4h) demonstrates that the resistance distribution at 0.2 V in both the high resistance state (HRS) and low resistance state (LRS) is concentrated. Furthermore, Figure 4i illustrates the stability of the fabricated device at 0.2 V in both the ON and OFF states. The current for both conditions shows no significant deterioration over a measurement period of more than 3 h. This result suggests that the memory device exhibits strong stability and a potentially low misreading rate. The reliability and stability of flash-type memory devices are closely related to their retention capacity and read cycle count. Cycling endurance tests were conducted to evaluate the reliability of the rewritable PFTC-g-RGO memory device (Figure 4j). In the first ten switching cycles, the device was subjected to +3 V voltage for a duration of 10 μs, followed by a 10 μs pause at 0 V, then switched to −3 V for 10 μs, and finally had another 10 μs pause at 0 V. The resistance levels in both the ON and OFF states remained stable even after 200 cycles of switching. Additionally, a series of programs were developed to simulate the device’s environment without an external power source. These programs utilized short pulse interval times (20 μs) and low pulse voltages (0.2 V) to evaluate the stability of the ON and OFF states. Figure 4k demonstrates that the device maintained its ON/OFF state throughout a test period of 1.0 × 10^5^ s after initially being switched under an externally applied voltage. Furthermore, Figure 4l swift and repeatable writing/erasing response of the device.

**Figure 5:**
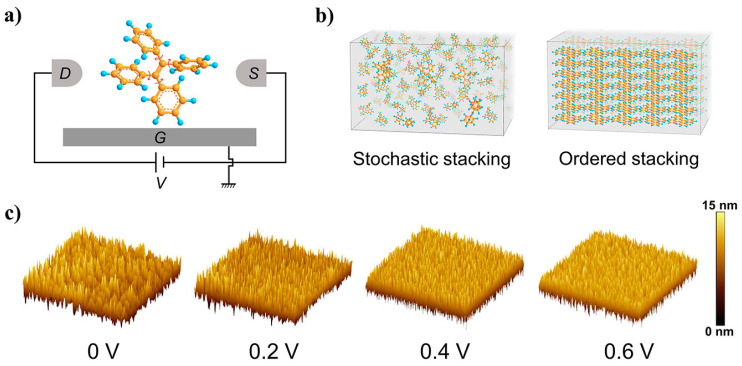
(**a**) Schematic diagram of a tetraphenylene molecule rotating under an electric field. (**b**) Schematic diagrams of the tetraphenylene molecules stacked in space. (**c**) AFM pictures of the PFTC-*g*-RGO film with a 4 × 4 μm^2^ scanning area at different voltages.

Due to the van der Waals force and strong π–π stacking interaction, RGO sheets tend to aggregate into multilayer structures or aggregates, resulting in their poor solubility or dispersion stability in water and organic solvents. This limitation hinders their practical application. To overcome this, we enhanced the solubility of RGO in organic solvents through polymer grafting. This modification allows for easy spin coating to prepare devices. Additionally, to reduce the π–π stacking interaction, we introduced tetraphenylethylene into the RGO preparation process. This effectively suppresses the interlayer cohesive energy, facilitating the separation of RGO sheets from each other. The molecular motion of the tetraphenylene was suppressed by electrical vibrational coupling. Figure 5a shows the voltage applied to a single tetraphenylene molecule, which is difficult to achieve in practice. Our designed polymer endowed tetraphenylene with a side chain. The side chain favored vibrations within the tetraphenylene molecule that might not be conducive to an ordered stacking of molecules. Figure 5b displays two extreme stackings of tetraphenylene molecules in space. Due to the excellent solubility of the material, a uniform film was easily spin-coated. As shown in Figure 5c, the surface roughness of the PFTC-*g*-RGO film without an applied voltage was 1.83 nm. To explore the changes in the surface roughness of the PFTC-*g*-RGO film after the vibration of the tetraphenylene molecules was damped, we applied different voltages to the film and performed in situ AFM characterization. As shown in Figure 5c, the surface roughness of the PFTC-*g*-RGO film decreased as the voltage increased. This phenomenon might be attributed to the effect of the applied voltage on the motion of the tetraphenylene molecules. At low applied voltages, the tetraphenylene side groups on the polymer backbone were initially randomly oriented. After the voltage was applied to the film, a large portion of the tetraphenylene groups underwent a conformational change, generating more regular π–π stacking regions between adjacent tetraphenylene groups, which enhanced the charge transport and generated a high conductivity state.

## 3. Materials and Methods

### 3.1. Sample Preparation

All of the chemical raw materials were obtained from Aldrich and directly used. Organic solvents were cleaned, dried, and distilled under dry nitrogen. All anhydrous and oxygen-free reactions were conducted using conventional Schlenk methods in a dry nitrogen environment. Monomers 2, 6, poly [9,9′-bis(6′′-bromohexyl) fluorene-alt-3,6-(9-(4-(1,2,2-triphenylvinyl) phenyl) carbazole)] (PBFTC), and PAFTC were synthesized following the steps outlined in the literature [39,40]. Detailed information on the synthesis method is provided in the Supplementary Information.

### 3.2. Reduction of GO

GO was prepared from powdered graphite following Hummers’ method [41]. Approximately 3.0 g of graphite was added to 125 mL of H_2_SO_4_ (98%) in an ice bath. KMnO_4_(1.5 g) was dissolved in water and vigorously stirred for 30 min at a constant temperature of the mixture solution (0 °C), and then the mixture was carefully and slowly added to the solution. The resulting solution was diluted with 250 mL of deionized water; then the mixture was slowly warmed to 35 °C and stirred for 2 h. Afterward, 75 mL of deionized water was added to the mixed solution. An aliquot of H_2_O_2_ (30%, 15 mL) was added to the solution to react with an excess KMnO_4_. After the mixture was filtered, the solid was washed with HCl (3%, 25 mL) and deionized water (25 mL), dried through lyophilization for 48 h, and then 4.1 g of GO was obtained.

Precisely, 100 mg of GO was ultrasonically dispersed into 200 mL of deionized water to form a homogeneous GO aqueous solution. A 2 mL hydrazine hydrate mixture containing 64.2 mmol was heated to 95 °C. After the mixture was vigorously agitated for 12 h, the resultant suspension was sifted through a filter paper of 45 μm in diameter. Then, obtained solid was washed with ethanol and deionized water multiple times, and the black solid was freeze-dried for 24 h, yielding 87 mg of RGO.

### 3.3. Synthesis of PFTC-g-RGO

RGO (20 mg) was ultrasonically dispersed in NMP (20 mL) for 30 min. PAFTC (200 mg) was dissolved in NMP (15 mL) and stirred with a stirring bar under an inert atmosphere. Then, the RGO dispersion was transferred to the polymer solution. The mixed solution was stirred at 160 °C for 3 d. After the mixed solution was cooled to room temperature and filtered, the residual solution was dialyzed against deionized water (MW cutoff, 3.5 kDa) for 3 d, and water was replaced with fresh water four times a day. The final product (PFTC-*g*-RGO) was obtained through filtration and then freeze-dried for 24 h. Yield: 203 mg.

### 3.4. Device Preparation and Characterization

The ITO glass substrate was subjected to meticulous pre-cleaning for 15 min in an ultra-sonic bath. After that, a solution of PFTC-g-RGO in NMP (5 mg/mL) was spin-coated onto the ITO glass substrate using 100 μL for 10 s at 700 rpm followed by 45 s at 1400 rpm. The substrate was then vacuum dried, and the ITO glass substrate was removed. Aluminum electrodes were evaporated onto the surface of the PFTC-g-RGO film using a vacuum coating instrument at a pressure of 10^−7^ Torr. All performance tests were conducted on a Keithley 4200 without device packaging and under normal temperature and pressure conditions.

## 4. Conclusions

PAFTC, a new soluble aggregation-induced emission polymer material, was used to chemically modify RGO to obtain PFTC-*g*-RGO. Transmission electron microscopy, FT-IR, and the Raman spectrum revealed that the polymer was successfully grafted onto the surface of the functionalized reduced graphene oxide. XPS analysis revealed that the carbon–nitrogen bond and the tertiary amine were present in the novel materials. The UV/Vis absorption, fluorescence, and electron paramagnetic resonance spectra of PFTC-*g*-RGO revealed the occurrence of intramolecular charge transfer. The outstanding properties of the manufactured Al/PFTC-*g*-RGO/ITO devices, including a low “turn OFF” voltage, high ON/OFF current ratios of up to 10^3^, great endurance over 200 switching cycles, and lengthy retention durations of over 10^4^ s, showed that the devices have good memory characteristics. The “turn ON” voltage had a relatively small range between 0.585 and 0.605 V, while the “turn off” voltage had a small range between −2.315 and −2.290 V, indicating a high reproducibility of the Al/PFTC-*g*-RGO/ITO devices.

## Figures and Tables

**Table 1:** Memory performance comparison with resistive, nonvolatile memory base on graphene and its derivatives.

Active Layer	ON/OFF Ratio	ON (V)	OFF (V)	Ref
GO-PFCz	10^3^	−1.30	3.30	31
GO-PTHF	10^3^	−1.20	2.80	32
RGO-PFTPA	10^3^	−1.40	1.65	33
PAE-*g*-RGO	10^3^	−2.80	3.17	34
CNT/PVK	10^3^	−1.85	2.90	35
PDDF-*g*-MWNTs	10^4^	2.07	−2.45	36
PFTC-*g*-RGO	10^3^	0.60	−2.30	This work

## Data Availability

Not applicable.

## Supplementary Materials

The following supporting information can be downloaded at: https://www.mdpi.com/article/10.3390/molecules28176271/s1, Scheme S1: Synthetic routes of PAFTC; Figure S1: (a) IR spectra of MWNTs, PAFTC and PFTC-g-MWNTs. (b) Raman spectra of the samples (lex = 785 nm). (c)TGA curves of MWNTs, PAFTC and PFTC-g-MWNTs. (d) XPS wide scan spectra of PFTC-g-MWNTs, (e) C1s and (f) N1s core-level spectra of PFTC-g-MWNTs. (g) UV/Vis spectra of MWNTs, PAFTC, PFTC-g-MWNTs. (h) UV/Vis spectra of PFTC-g-MWNTs at a range of concentrations from 5 to 50 μg∙mL^−1^. Inset shows the correlation curve of PFTC-g-MWNTs at 350 nm to the concentration. (i) Photoluminescence spectra of PFTC-g-MWNTs at different solvents (lex = 350 nm). (j) The cyclic voltammetry curve of the PAFTC film. (k) The cyclic voltammetry curve of the PFTC-g-MWNTs film. (l) Current-voltage performance of the Al/PFTC-g-MWNTs/ITO device; Figure S2: Ex-perimental data and fitted line of I-V characteristics for the as-fabricated Al/PFTC-g-RGO/ITO device in the OFF state (a) and ON state (b).

## Sample Availability

Not available.

## References

[1] AGeim K., Novoselov K.S. (2009). The rise of graphene. Nanoscience and Technology.

[2] Chen Y., Zhang B., Liu G., Zhuang X., Kang E.-T. (2012). Graphene and its derivatives: Switching ON and OFF. Chem. Soc. Rev..

[3] Chen Y., Bai T., Dong N., Fan F., Zhang S., Zhuang X., Sun J., Zhang B., Zhang X., Wang J. (2016). Graphene and its derivatives for laser protection. Prog. Mater. Sci..

[4] Jolly A., Miao D., Daigle M., Morin J.-F. (2020). Emerging Bottom-Up Strategies for the Synthesis of Graphene Nanoribbons and Related Structures. Angew. Chem..

[5] Ahmed S., Yi J. (2017). Two-Dimensional Transition Metal Dichalcogenides and Their Charge Carrier Mobilities in Field-Effect Transistors. Nano-Micro Lett..

[6] Kausar A. (2017). Carbon nano onion as versatile contender in polymer compositing and advance application. Fuller. Nanotub. Carbon Nanostructures.

[7] Singh V., Joung D., Zhai L., Das S., Khondaker S.I., Seal S. (2011). Graphene based materials: Past, present and future. Prog. Mater. Sci..

[8] Ponraj J.S., Xu Z.-Q., Dhanabalan S.C., Mu H., Wang Y., Yuan J., Li P., Thakur S., Ashrafi M., Mccoubrey K. (2016). Photonics and optoelectronics of two-dimensional materials beyond graphene. Nanotechnology.

[9] Ma R., Zhou Y., Bi H., Yang M., Wang J., Liu Q., Huang F. (2020). Multidimensional graphene structures and beyond: Unique properties, syntheses and applications. Prog. Mater. Sci..

[10] Sagadevan S., Shahid M.M., Yiqiang Z., Oh W.-C., Soga T., Lett J.A., Alshahateet S.F., Fatimah I., Waqar A., Paiman S. (2021). Functionalized graphene-based nanocomposites for smart optoelectronic applications. Nanotechnol. Rev..

[11] Yang F., Song P., Xu W. (2020). The Applications of 2D Nanomaterials in Energy-Related Process. Adapting 2D Nanomaterials for Advanced Applications.

[12] Abbas A., Mariana L.T., Phan A.N. (2018). Biomass-waste derived graphene quantum dots and their applications. Carbon.

[13] Saeidi M., Lee M., Okello O.F.N., Choi S.-Y., Oh S.S., Simchi A. (2021). Ultrafast Graphitization and Reduction of Spongy Graphene Oxide by Low-Energy Electromagnetic Radiation to Boost the Performance and Stability of Carbon-Based Supercapacitors. ACS Appl. Energy Mater..

[14] Englert J.M., Dotzer C., Yang G., Schmid M., Papp C., Gottfried J.M., Steinrück H.-P., Spiecker E., Hauke F., Hirsch A. (2011). Covalent bulk functionalization of graphene. Nat. Chem..

[15] Toh S.Y., Loh K.S., Kamarudin S.K., Daud W.R.W. (2014). Graphene production via electrochemical reduction of graphene oxide: Synthesis and characterization. Chem. Eng. J..

[16] Chouhan A., Mungse H.P., Khatri O.P. (2020). Surface chemistry of graphene and graphene oxide: A versatile route for their dispersion and tribological applications. Adv. Colloid Interface Sci..

[17] Bai S., Shen X. (2012). Graphene–inorganic nanocomposites. RSC Adv..

[18] Khan Z.U., Kausar A., Ullah H., Badshah A., Khan W.U. (2016). A review of graphene oxide, graphene buckypaper, and polymer/graphene composites: Properties and fabrication techniques. J. Plast. Film. Sheeting.

[19] Karki N., Tiwari H., Tewari C., Rana A., Pandey N., Basak S., Sahoo N.G. (2020). Functionalized graphene oxide as a vehicle for targeted drug delivery and bioimaging applications. J. Mater. Chem. B.

[20] Huang X., Yin Z., Wu S., Qi X., He Q., Zhang Q., Yan Q., Boey F., Zhang H. (2011). Graphene-based materials: Synthesis, characterization, properties, and applications. Small.

[21] Chen D., Feng H., Li J. (2012). Graphene oxide: Preparation, functionalization, and electrochemical applications. Chem. Rev..

[22] Stergiou A., Cantón-Vitoria R., Psarrou M.N., Economopoulos S.P., Tagmatarchis N. (2020). Functionalized graphene and targeted applications–Highlighting the road from chemistry to applications. Prog. Mater. Sci..

[23] Alhazmi H.A., Ahsan W., Mangla B., Javed S., Hassan M.Z., Asmari M., Al Bratty M., Najmi A. (2022). Graphene-based biosensors for disease theranostics: Development, applications, and recent advancements. Nanotechnol. Rev..

[24] Babu S.S., Praveen V.K., Ajayaghosh A. (2014). Functional π-gelators and their applications. Chem. Rev..

[25] Cao Y., Fu Y., Li D., Zhu C., Zhang B., Chen Y. (2019). Organophosphorus-based polymer covalently functionalized reduced graphene oxide: In-situ synthesis and nonvolatile memory effect. Carbon.

[26] Kagatikar S., Sunil D. (2019). Aggregation-induced emission of azines: An up-to-date review. J. Mol. Liq..

[27] Zhou L., Meng W., Sun L., Du L., Xuan X., Zhang J. (2022). Molecular motions of a tetraphenylethylene-derived AIEgen directly monitored through in situ variable temperature single crystal X-ray diffraction. CrystEngComm.

[28] RBhosale S., Aljabri M., La D.D., Bhosale S.V., Jones L.A., Bhosale S.V. (2019). Tetraphenylethene Derivatives: A Promising Class of AIE Luminogens—Synthesis, Properties, and Applications, In Principles and Applications of Aggregation-Induced Emission.

[29] Islam M.M., Hu Z., Wang Q., Redshaw C., Feng X. (2019). Pyrene-based aggregation-induced emission luminogens and their applications. Mater. Chem. Front..

[30] Yang J., Chi Z., Zhu W., Tang B.Z., Li Z. (2019). Aggregation-induced emission: A coming-of-age ceremony at the age of eighteen. Sci. China Chem..

[31] Zhang B., Liu Y.-L., Chen Y., Neoh K.-G., Li Y.-X., Zhu C.-X., Tok E.-S., Kang E.-T. (2011). Nonvolatile Rewritable Memory Effects in Graphene Oxide Functionalized by Conjugated Polymer Containing Fluorene and Carbazole Units. Chem.–A Eur. J..

[32] Zhuang X., Chen Y., Wang L., Neoh K.-G., Kang E.-T., Wang C. (2014). A solution-processable polymer-grafted graphene oxide derivative for nonvolatile rewritable memory. Polym. Chem..

[33] Zhang B., Chen Y., Liu G., Xu L.-Q., Chen J., Zhu C.-X., Neoh K.-G., Kang E.-T. (2012). Push–Pull archetype of reduced graphene oxide functionalized with polyfluorene for nonvolatile rewritable memory. J. Polym. Sci. Part A Polym. Chem..

[34] Sun S., Zhuang X., Liu B., Wang L., Gu L., Song S., Zhang B., Chen Y. (2016). In Situ Synthesis and Characterization of Poly(aryleneethynylene)-Grafted Reduced Graphene Oxide. Chem.–A Eur. J..

[35] Liu G., Ling Q.-D., Teo E.Y.H., Zhu C.-X., Chan D.S.-H., Neoh K.-G., Kang E.-T. (2009). Electrical Conductance Tuning and Bistable Switching in Poly(N-vinylcarbazole)−Carbon Nanotube Composite Films. ACS Nano.

[36] Gua M., Zhao Z., Lie J., Liu G., Zhang B., El-Khouly M.E., Chen Y. (2021). Conjugated polymer covalently modified multi-walled carbon nanotubes for flexible nonvolatile RRAM devices. Eur. Polym. J..

[37] Cao L., Sun Q., Wang H., Zhang X., Shi H. (2015). Enhanced stress transfer and thermal properties of polyimide composites with covalent functionalized reduced graphene oxide. Compos. Part A Appl. Sci. Manuf..

[38] Wróbel D., Graja A. (2011). Photoinduced electron transfer processes in fullerene–organic chromophore systems. Coord. Chem. Rev..

[39] Liu B., Bazan G.C. (2006). Synthesis of cationic conjugated polymers for use in label-free DNA microarrays. Nat Protoc..

[40] Dong W., Fei T., Palma-Cando A., Scherf U. (2014). Aggregation induced emission and amplified explosive detection of tetraphenylethylene-substituted polycarbazoles. Polym. Chem..

[41] Hummers W.S., Offeman R.E. (1958). Preparation of Graphitic Oxide. J. Am. Chem. Soc..

