# Supplementary material for: Fabrication of AIE Polymer-Functionalized Reduced Graphene Oxide for Information Storage
Source: Molecules. 2023 Aug 27;28(17):6271. doi: 10.3390/molecules28176271 (PMC10488735; doi:10.3390/molecules28176271)
Supplement: Supplementary file 1 [file molecules-28-06271-s001.zip › molecules-2573717-supplementary.pdf]

Supporting Information

Fabrication of AIE Polymer-Functionalized Reduced Graphene Oxide for Information Storage

Kai Gao [1,2], Wei Li [2], Xiaoyang Wang [3], Sai Sun [1,*] and Bin Zhang [2,*]

1 Sinopec Shanghai Research Institute of Petrochemical Technology, Shanghai 201208, China
2 Key Laboratory for Advanced Materials and Joint International Research Laboratory of Precision Chemistry and Molecular Engineering, School of Chemistry and Molecular Engineering, East China University of Science and Technology, Shanghai 200237, China
3 Guangxi Key Laboratory of Information Material, Engineering Research Center of Electronic Information Materials and Devices, School of Material Science and Engineering, Guilin University of Electronic Technology, Guilin 541004, China
* Correspondence: suns.sshy@sinopec.com (S.S.); zhangbin@ecust.edu.cn (B.Z.)

Experimental section

Measurements and Instrument

All chemicals were purchased from Aldrich (Shanghai, China) and used without further purification. The ^{1}H nuclear magnetic resonance (^{1}H NMR) spectra were performed at 400 MHz on a Bruker 400 AVANCE III spectrometer with chloroform as solvent and tetramethylsilane (TMS) as a reference for the chemical shifts. Weight-average (M_w) and number-average (M_n) molecular weights were determined using Waters 2690 gel permeation chromatography (GPC) using polystyrene standards eluted with tetrahydrofuran (THF, 1 mL/min). UV-Visible absorption spectra of the polymer were obtained in a diluted solution on a Shimadzu UV-2450 spectrophotometer. Steady-state fluorescence spectra of the polymer devices were recorded with an Andor SR303i-A/DU420A-BVF spectrofluorometer. Cyclic voltammetry (CV) measurements were measured in an electrolyte solution of tetrabutylammonium hexafluorophosphate (n-Bu_4NPF_6) in acetonitrile (0.1 M) under an argon atmosphere, using platinum gauze and Ag/AgCl as the counter and reference electrodes respectively. A typical scan rate of 50 mV/s was used during the CV measurements.

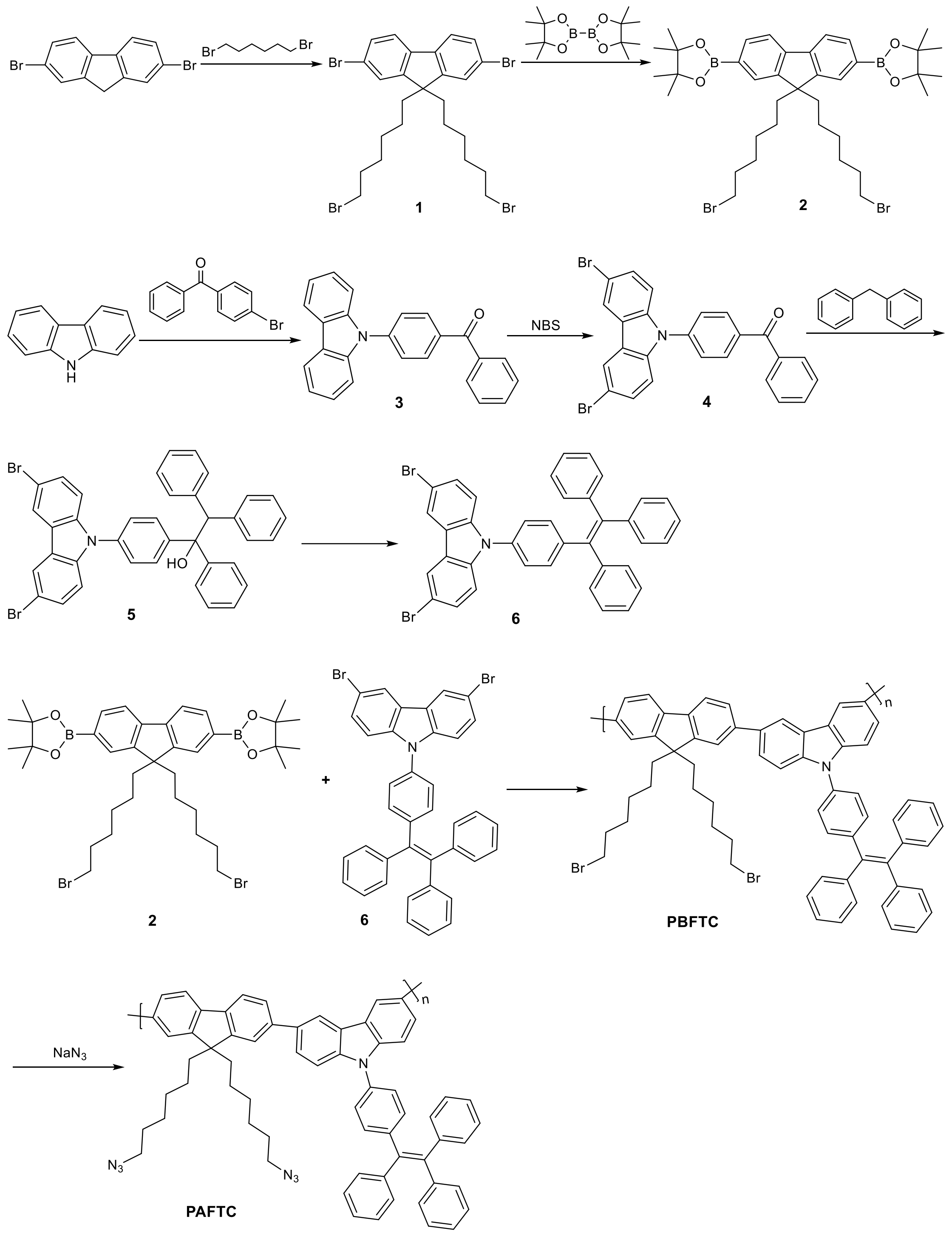

Scheme S1. Synthetic routes of PAFTC.

Synthesis of compound 1: tetrabutylammonium bromide (900 mg, 2.79 mmol) and aqueous sodium hydroxide (250 mL, 50%wt) were added to a 500 mL round-bottom flask and stirred using a stirring bar. After the mixture was heated to 75°C, 1,6-dibromohexane (67.8 g, 277.9 mmol) and 2,7-dibromofluorene (9.72 g, 30 mmol) were added to the mixture and stirred for 2 h at 75°C. Afterward, the mixture was cooled to room temperature and extracted with CH_2Cl_2.

The combined organic layer was washed with deionized water and aqueous HCl (0.1M) three times. Then, the washed sample was dehydrated with anhydrous $MgSO_4$. After the filtration and solvent evaporation of the sample, a vacuum pump was used to distill off excess 1,6-dibromohexane under reduced pressure. Then, the crude product was purified with silica-gel column chromatography (CH_2Cl_2/petroleum ether =1:6) to produce compound 1 as a white solid. Yield: 13.8 g (70%). ^{1}H NMR (400 MHz, $CDCl_3$): 7.61–7.43 (m, 6H), 3.30–3.27 (t, 4H), 1.9 (t, 4H), 1.72–1.64 (m, 4H), 1.24–1.07 (m, 8H), 0.62 (m, 4H).

Synthesis of Compound 2: compound 1 (6.5 g, 10 mmol), bis(pinacolato)diboron (6.0 g, 24 mmol), and potassium acetate (7.0 g, 71 mmol) were dissolved in dioxane (100 ml) under N_2. Then, the mixed solution was bubbled for 30 min; [1,1-bis(diphenylphosphino)ferrocene]dichloro palladium (II) (0.5 g) was added and stirred at 85°C for 24 h with a stirring bar. After the mixed solution was cooled to room temperature, the dioxane was evaporated under reduced pressure. Then, deionized water (50 mL) was added to the sample and extracted with CH_2Cl_2 (100 mL*3). The combined organic layer was washed with deionized water and dehydrated with anhydrous $MgSO_4$. After the filtration and solvent evaporation of the sample, the crude product was purified by silica-gel column chromatography (CH_2Cl_2/petroleum ether =1:6), and compound 2 was obtained as a white solid. Yield: 3.4 g (45%). ^{1}H NMR (400 MHz, $CDCl_3$): 7.83–7.70 (m, 6H), 3.23 (t, 4H), 2.03–1.98 (m, 4H), 1.60 (q, 4H), 1.39 (s, 24H), 1.17–1.12 (q, 4H), 1.07–1.01 (q, 4H), and 0.57–0.52 (m, 4H).

Synthesis of Compound 3: 4-bromobenzophenone (5.01 g, 30 mmol), carbazole (8.613 g, 33 mmol), $Pd(OAc)_2$ (0.336 g, 1.5 mmol), $P(t\text{-}Bu)_3$ (3.0 mL, 3.0 mmol), K_2CO_3 (120 mmol, 16.58 g), and toluene (100 mL) were placed into to a 250 mL two-necked round-bottom flask and stirred with a stirring bar under N_2 atmosphere and heated to 120°C for 48 h. After the

mixed solution was cooled to room temperature, it was added to cold water (500 mL) and stirred for another 6 h. Then, the mixture was extracted with CH_2Cl_2. The combined organic layer was washed with water and dehydrated with anhydrous $MgSO_4$. After the filtration and solvent evaporation of the sample, the crude product was purified using silica-gel column chromatography (CH_2Cl_2/petroleum ether =1:4) to afford Compound 3 as a gray solid. Yield: 7.4 g (71%).^{1}H NMR (400 MHz, DMSO): 8.28 (d, 2H), 8.05 (d, 2H), 7.89 (d, 2H), 7.86 (d, 2H), 7.77 (t, 1H), 7.64–7.56 (m, 4H), 7.50–7.46 (m, 2H), and 7.36–7.32 (m, 2H).

Synthesis of Compound 4: Compound 3 (5.7 g, 17.81 mmol) was dissolved in DMF (60 mL) and placed in an ice bath under an N_2 atmosphere. N-bromosuccinimide (7.13 g, 39.18 mmol) was dissolved in DMF (30 mL) and added dropwise to the solution. The mixture was stirred for 12 h at room temperature. Afterward, the mixture was added to cold water (500 mL) and then formed compound 4, and the light yellow precipitate (compound 4) was collected through filtration. Yield: 7.4 g (82%).^{1}H NMR (400 MHz, DMSO): 8.62 (d, 2H), 8.04 (t, 2H), 7.87–7.86 (m, 2H), 7.76–7.71 (m, 3H), 7.65–7.60 (m, 4H), and 7.52 (d, 2H).

Synthesis of Compound 6: diphenylmethane (2.4 g, 14.26 mmol) and THF (40 mL) were placed into a 250 mL two-necked round bottom flask. Then, the two-necked round bottom flask containing the mixed solution was placed in an ice bath under the N_2 atmosphere. N-butyllithium (5.2 mL, 12.01 mmol) was dropwise added into the solution and stirred at 0°C for 1 h. Then, the solution of compound 4 (6.0 g, 11.88 mmol) in THF (40 mL) was added. The mixture was stirred at 0°C for another 0.5 h and at room temperature for 24 h. The reaction was quenched by adding the saturated solution of ammonium chloride, extracted with CH_2Cl_2, and dehydrated with anhydrous $MgSO_4$. After the filtration and solvent evaporation of the sample, the crude product of compound 5 was obtained as a gray solid. All the solid and p-toluenesulfonic acid (10 g) were dissolved in toluene (100 mL) and heated to reflux for

12 h. After the mixed solution was cooled to room temperature, the solution was washed with saturated NaCl solution and dehydrated with anhydrous $MgSO_4$. After the dehydrated solution was filtered and solvent-evaporated, the resulting product was obtained with silica-gel column chromatography (CH_2Cl_2/petroleum ether = 1:3). Yield: 3.6 g (46%). ^{1}H NMR (400 MHz, DMSO): 8.57 (s, 2H), 7.61–7.59 (d, 2H), and 7.25–7.04 (m, 21H).

Synthesis of PBFTC: compound 2 (758 mg, 1 mmol), compound 6 (655 mg, 1 mmol), and toluene (4 mL) were added in a 50 mL Schlenk flask under an N_2 atmosphere. K_2CO_3 (276 mg, 2 mmol) was dissolved in deionized water (1 mL) and added to the above mixed solution. After the mixed solution was bubbled with N_2 for 30 min, $Pd(PPh_3)_4$ (20 mg) was added to the mixed solution and vigorously stirred at 85°C for 48 h. Then, the mixed solution was cooled to room temperature, dropwise added to methanol (400 mL), and stirred with a stirring bar to form precipitates. The precipitated material was filtrated and redissolved in toluene. Then, the filtrate solution was dropwise added to methanol and filtrated again. The resulting materials (PBFTC) were washed with acetone and vacuum dried for 24 h at 60°C. Yield: 447 mg (45%). M_w = 13464, M_w/M_n = 1.45; ^{1}H NMR (400 MHz, DMSO): 8.46 (s, 4H), 7.73–7.46 (m, 4H), 7.28–7.01 (m, 23H), 3.52 (t, 4H), 1.83–1.70 (m, 8H), and 1.29–1.16 (m, 12H).

Synthesis of PAFTC: PBFTC (493 mg, 0.5 mmol of its repeat unit) was dissolved in anhydrous THF (30 mL) in a 250 mL round-bottom flask and stirred with a stirring bar. NaN_3 (130 mg, 2 mmol) was dissolved in DMF (30 mL), ultrasonically stirred for 30 min, and then added to the PBFTC solution. The mixture was heated to reflux for 48 h. After the mixed solution was cooled to room temperature and solvent-evaporated with THF, the residual solution was dialyzed against deionized water (MW cutoff, 3.5 kDa) for 3 d, and the used water was replaced with freshwater four times a day during the dialysis. The final product (PAFTC) was filtered and freeze-dried for 24 h. Yield: 429 mg (94%). M_w = 16434, M_w/M_n =

1.82; ^{1}H NMR (400 MHz, DMSO): 8.51 (s, 4H), 7.90–7.73 (m, 4H), 7.46–7.20 (m, 23H), 1.82 (t, 4H), 1.49 (t, 4H), 1.30–1.21 (m, 16H).

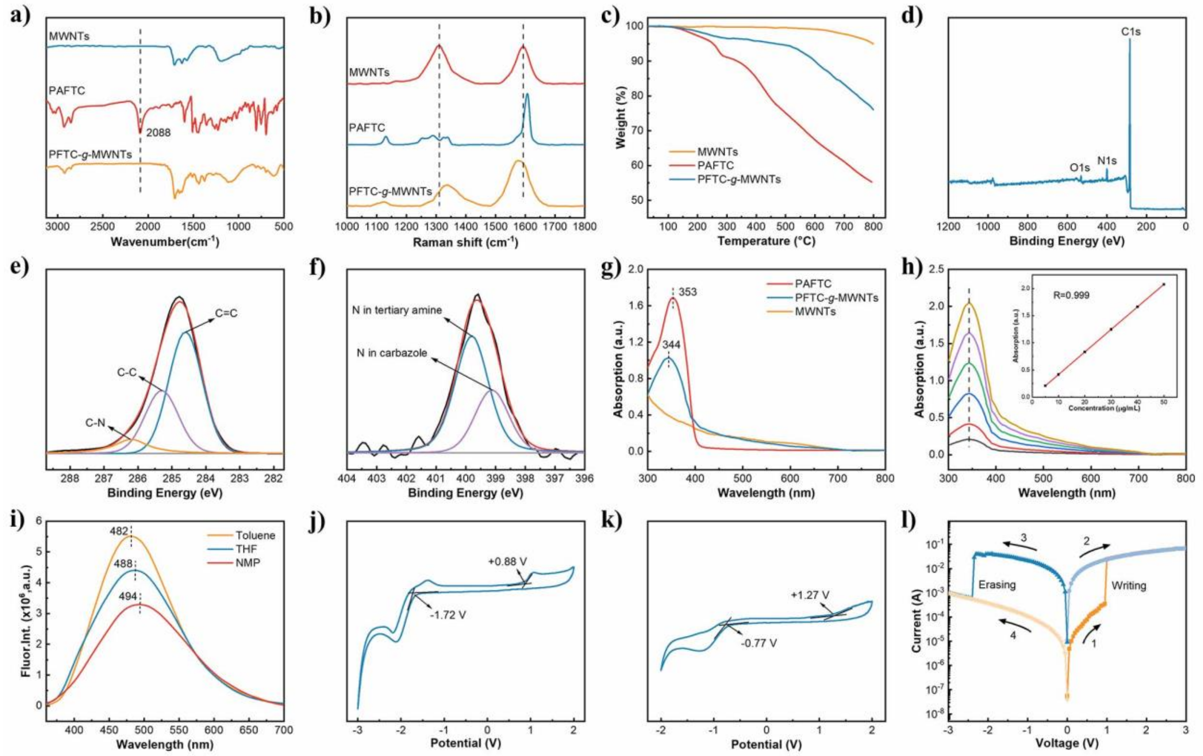

Figure S1. (a) IR spectra of MWNTs, PAFTC and PFTC-*g*-MWNTs. (b) Raman spectra of the samples (λ_{ex}=785nm). (c)TGA curves of MWNTs, PAFTC and PFTC-*g*-MWNTs. (d) XPS wide scan spectra of PFTC-*g*-MWNTs, (e) C1s and (f) N1s core-level spectra of PFTC-*g*-MWNTs. (g) UV/Vis spectra of MWNTs, PAFTC, PFTC-*g*-MWNTs. (h) UV/Vis spectra of PFTC-*g*-MWNTs at a range of concentrations from 5 to 50 $\mu g\cdot mL^{-1}$. Inset shows the correlation curve of PFTC-*g*-MWNTs at 350 nm to the concentration. (i) Photoluminescence spectra of PFTC-*g*-MWNTs at different solvents (λ_{ex}=350nm). (j) The cyclic voltammetry curve of the PAFTC film. (k) The cyclic voltammetry curve of the PFTC-*g*-MWNTs film. (l) Current-voltage performance of the Al/PFTC-*g*-MWNTs/ITO device.

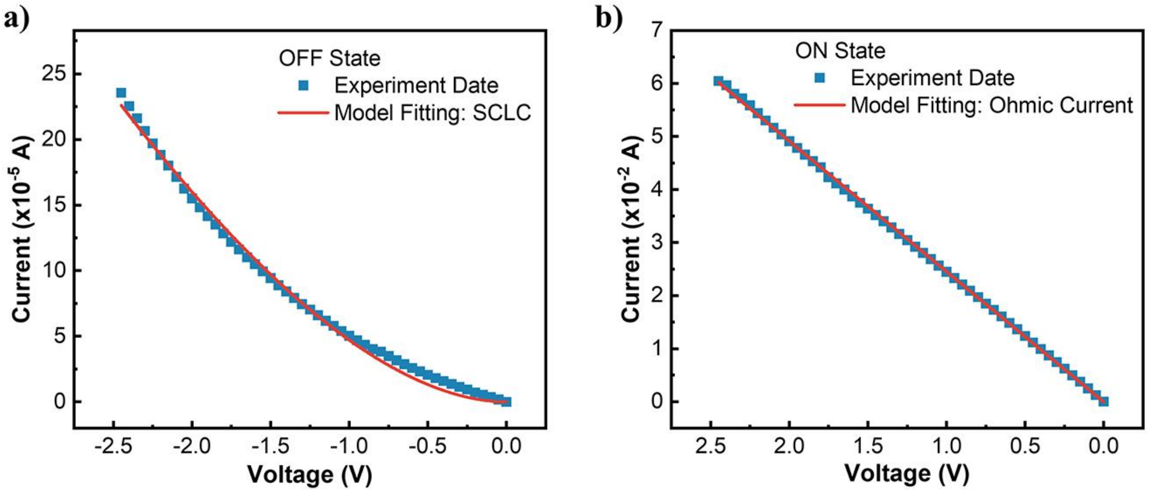

Figure S2. Experimental data and fitted line of I-V characteristics for the as-fabricated Al/PFTC-*g*-RGO/ITO device in the OFF state (a) and ON state (b).

Without input voltage, the electrons in the system were stable, implying that the fabricated Al/PFTC-*g*-RGO/ITO device was in the OFF mode. The OFF mode current can be fitted by a space-charge-limited current (SCLC) model (**Figure S2a**):

$$J = A\mu\varepsilon\varepsilon_0 \frac{V^2}{d^3} \exp\{\frac{0.891}{kT}(\frac{q^3 V}{\pi\varepsilon\varepsilon_0 d})^{1/2}\} \quad (1)$$

where J, A, μ, $\varepsilon\varepsilon_0$, d, V, k, T, and q are current density, positive constant, the mobility of charge carriers, the absolute permittivity of the complex, the thickness of the film, voltage, the Boltzmann constant, the ambient temperature, and the absolute value of the unit electronic charge (1.6×10^{-19} C), respectively. The ON mode current can be fitted by an Ohmic current model (**Figure S2b**)

$$J = B\frac{qnv}{d} \quad (2)$$

where B is a positive constant, and n is the density of charge carriers. The intensive electron delocalization in RGO can effectively stabilize the conductive CT state, resulting in the nonvolatile nature of the ON mode.
